# National control programs for scabies: Experiences from Fiji and Solomon Islands

**DOI:** 10.1371/journal.pntd.0013874

**Published:** 2026-02-05

**Authors:** Sarah Andersson, Matthew Parnaby, Susanna Lake, Julie Zinihite, Vinaisi Bechu, Ripeka Kaurasi, Aalisha Sahukhan, Hannah Andrews, Tessa Hughes, Lucia Romani, Daniel Engelman, John Kaldor, Aminiasi Tavui, Oliver Sokana, Andrew Steer

**Affiliations:** 1 World Scabies Program, Murdoch Children’s Research Institute, Melbourne, Victoria, Australia; 2 Tropical Diseases Research Group, Murdoch Children’s Research Institute, Melbourne, Victoria, Australia; 3 Centre for Disease Control, Ministry of Health and Medical Services, Suva, Fiji; 4 Kirby Institute, University of New South Wales, Sydney, New South Wales, Australia; 5 Neglected Tropical Diseases Unit, Ministry of Health and Medical Services, Honiara, Solomon Islands; 6 Department of Paediatrics, University of Melbourne, Melbourne, Victoria, Australia; George Washington University Medical Center, UNITED STATES OF AMERICA

## Abstract

**Introduction:**

The most recent World Health Organization roadmap for neglected tropical diseases sets a target for countries to control scabies through several interventions, including mass drug administration in endemic areas where prevalence is 10% or greater using oral ivermectin and topical scabicides. This report documents the experiences and lessons learned from the first two countries in the world, Fiji and Solomon Islands, to implement national ivermectin-based mass drug administration for scabies. By identifying key challenges, this article aims to inform the global community as efforts are established to reach these targets.

**Program description:**

The World Scabies Program was established by the Murdoch Children’s Research Institute in 2019, with the Kirby Institute at the University of New South Wales and the Ministries of Health in Fiji and Solomon Islands as key partners. The Program aims to translate research findings from studies of ivermectin-based mass drug administration into national scabies control programs in Fiji and Solomon Islands.

**Lessons learned:**

The Program adapted to meet several challenges. The COVID-19 pandemic restricted travel by Program staff, necessitating greater local autonomy. Addressing local misconceptions of scabies improves uptake and health worker motivation. Integrating with other neglected tropical diseases and health programs improved affordability and acceptability of the Program. New strategies are required to reach urban populations, and pragmatic dosing options across all ages would increase feasibility.

**Conclusions:**

The implementation of scabies mass drug administration in highly endemic areas has the potential to lead to substantial improvements in health outcomes if large populations are reached. The impact of the mass drug administration campaigns in Fiji and Solomon Islands remains to be fully evaluated, but we have already learnt critical lessons to inform future efforts towards scabies control.

## Author summary

Human scabies is a highly prevalent skin disease, with an estimated global prevalence of over 200 million people, with an increased burden in tropical regions, particularly in children and young adults, and in areas with poor socioeconomic conditions. Persistently high scabies prevalence has long-term health and social consequences for individuals and their communities, and financial consequences to health systems in managing the complications of scabies. The most recent WHO roadmap for NTDs sets a target for countries to control scabies through a number of interventions including mass drug administration (MDA, also called preventive chemotherapy) in endemic areas, where prevalence is 10% or greater, using oral ivermectin and topical scabicides. This report aims to contribute to the overall evidence for public health control of scabies by documenting the experiences and lessons learned from the first two countries in the world, Fiji and Solomon Islands, to implement national ivermectin-based MDA for scabies in a non-research setting.

## Introduction

Human scabies is a common skin disease, with an estimated global prevalence of over 200 million people [1]. Scabies is found in every country but causes the greatest burden of disease in resource-poor, tropical settings. In 2017, the World Health Organization (WHO) recognized scabies as a neglected tropical disease (NTD) [2]. The most recent roadmap for NTDs, “Ending the Neglect to Attain the Sustainable Development Goals: a Road Map for NTDs 2021–2030,” sets a target for countries to control scabies through a number of interventions including mass drug administration (MDA, also called preventive chemotherapy) in endemic areas, where prevalence is 10% or greater, using oral ivermectin and topical scabicides [3]. This recognition of scabies as a NTD and the recommendation to use public health interventions such as MDA to control scabies is the result of a growing evidence base of both the impact of scabies and the effectiveness of MDA. Persistently high scabies prevalence has long-term health and social consequences for individuals and their communities and financial consequences to health systems in managing the complications of scabies [2].

Scabies is caused by the parasitic mite *Sarcoptes scabiei* var. *hominis* and is a highly contagious infection spread predominantly via skin-to-skin contact. Transmission of scabies commonly occurs among household members where there is overcrowding and poverty, and highest prevalence is observed in low-resource communities [4]. The mite burrows under the skin and deposits eggs resulting in an immune response that causes intense itching. Itch and scratch can lead to broken skin which facilitates bacterial skin infection, most commonly impetigo [5]. Serious complications of bacterial skin infection include more widespread skin and soft tissue infections such as cellulitis and necrotizing fasciitis, bloodstream infection, and immune-mediated diseases. Scabies infection and subsequent acute post-streptococcal glomerulonephritis in childhood has been proposed as a major contributor to chronic kidney disease later in life [6]. A growing body of evidence also implicates impetigo caused by *Streptococcus pyogenes* in the pathogenesis of rheumatic fever and rheumatic heart disease [6–8]. Furthermore, scabies by itself causes intense itch that can lead to work or school absenteeism because of lack of sleep, and scabies can also contribute to social stigma [9].

Preventive chemotherapy (or MDA) is a public health strategy recommended for several NTDs for the past twenty to thirty years [10]. This strategy has contributed to substantial progress in reducing the burden of several NTDs; lymphatic filariasis has been eliminated as a public health problem in 17 countries, trachoma in 10, and onchocerciasis in four [3]. MDA involves administering safe and effective medicines to everyone in a community, regardless if they have signs of disease. Based on the success of this strategy for other diseases, studies have been conducted over the last decade to determine if MDA could be effective and safe to control or even eliminate scabies as a public health problem [11].

Several of these studies were conducted in Pacific Island Nations, namely Fiji and Solomon Islands, where prevalence of scabies, impetigo, and rheumatic heart disease are high [5]. This research demonstrated that in highly endemic areas MDA can substantially reduce prevalence of scabies and impetigo, and in one study also reduce clinic presentations and hospitalisations for soft tissue infections. In Fiji, a study using ivermectin-based MDA reduced community prevalence of scabies by 94% one-year after the MDA intervention (prevalence 32%, to less than 2%) compared to 49% reduction in the standard care control arm (36.6%–18.8%) [12]. A larger single-arm study of ivermectin-based MDA (integrated with trachoma control) among 26,000 people in Solomon Islands demonstrated a 88% reduction in scabies prevalence (18.7%–2.3%) [13]. An even larger single-arm study of ivermectin-based MDA (integrated with LF control) among 135,000 people in Fiji demonstrated that ivermectin-based MDA reduced hospitalizations for skin and soft tissue infections by 17% in the year after the intervention compared to baseline [14]. All three studies demonstrated reductions in community impetigo prevalence between 60% and 88%.

The WHO convened an informal consultation on a scabies control in 2019 to develop a framework and recommendations for countries to implement population-level control strategies for scabies [15]. The Framework recommends that preventive chemotherapy be considered in areas where the community prevalence of scabies infestation is ≥10% of the whole population. The Framework acknowledges that further evidence and experience are required to refine the strategies over time and to eventually develop formal guidelines.

The WHO NTD roadmap has a target for 25 countries to implement MDA in endemic areas by the end of 2030. This report aims to contribute to the overall evidence for public health control of scabies as the global community seeks to achieve these targets, by documenting the experiences and lessons learned from the first two countries in the world, Fiji and Solomon Islands, to implement national ivermectin-based MDA for scabies in a non-research setting.

## Program description

The World Scabies Program was established by the Murdoch Children’s Research Institute in 2019, with the Kirby Institute at the University of New South Wales as a key partner, to translate research findings from studies of ivermectin-based MDA into national scabies control programs. A critical component of the Program has been to understand the capacities and requirements to implement this approach at-scale in a non-research context. In partnership with their respective Ministries of Health, Fiji and Solomon Islands became the first two countries to scale the MDA strategy, within the context of historically high prevalence of scabies, involvement in formative scabies public health research, and commitment from their national governments to implement scabies control.

### Setting

**Fig 1:**
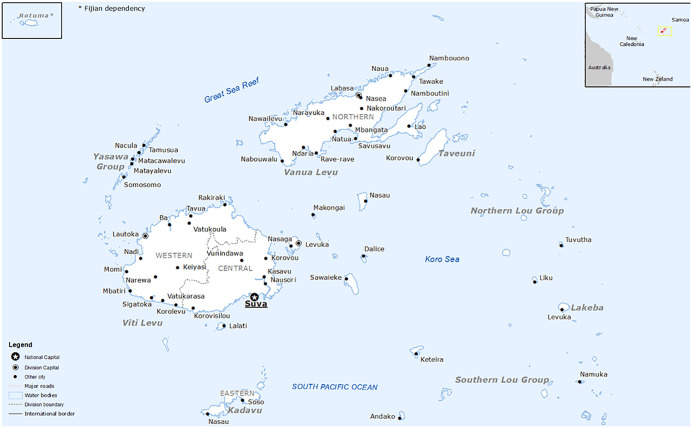
Map of Fiji. (The source of this map is https://commons.wikimedia.org/wiki/File:Fiji_Base_Map.png and is licensed under the Creative Commons Attribution 4.0 International (CC BY 4.0) License.).

**Fig 2:**
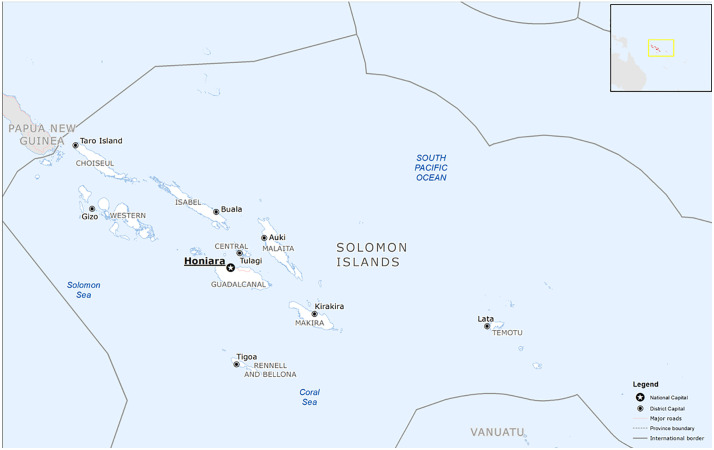
Map of Solomon Islands. (The source of this map is https://commons.wikimedia.org/wiki/File:Solomon_Islands_Base_Map.png and is licensed under the Creative Commons Attribution 4.0 International (CC BY 4.0) License.).

Fiji and Solomon Islands are independent Melanesian countries in the South Pacific. Fiji comprises over 300 islands, and has an estimated population of over 900,000, with the majority residing in urban areas on the two largest islands, Viti Levu and Vanua Levu (Fig 1) [16]. Solomon Islands comprises over 900 islands, and has an estimated population of over 721,000, most living across six major islands with 28% living in urban areas (Fig 2) [17].

Both countries have high prevalence of scabies. In Fiji, a national prevalence survey in 2007 observed that 23% of Fijians of all ages and 45% of children aged 5–9 years had scabies [18]. Similarly, in Solomon Islands, research surveys conducted over the last decade observed that 19% of the total population, and between 34% and 45% of children aged 5–9 years, had scabies [19].

### MDA implementation

In 2019, the World Scabies Program partnered with the Ministries of Health in Fiji and Solomon Islands to implement national ivermectin-based MDA for scabies. The design of MDA was aligned with the recommendations of the WHO Framework and drew from the experiences of MDA for other NTDs conducted in each country previously [15]. The WHO Framework recommendation is to treat all ages of the population by administering oral ivermectin for everyone except pregnant women, lactating mothers in the first week after birth, and children weighing <15 kg or <90 cm height. Individuals with a contraindication to ivermectin are offered permethrin 5% cream. The Framework recommends two doses of treatment given 7–14 days apart, as the initial treatment only kills mites (not eggs) and the second dose targets the newly hatched nymphs [2].

To ensure safe administration of ivermectin at national scale, pharmacovigilance mechanisms were employed, developed with the Ministry of Health in each country and aligned to WHO pharmacovigilance guidelines for mass drug administration [20]. Adverse events were monitored through passive surveillance, whereby health workers were trained to identify and document adverse events on MDA reporting forms and serious adverse event reporting forms. This included predefined criteria for immediate escalation of serious adverse events to the nearest health facility, and to the MDA focal person. Pharmacovigilance procedures were defined in collaboration with pharmacy departments in both countries. Active adverse events surveillance was implemented post-MDA during the coverage survey, which included questions on side effects and severity.

**Fig 3:**
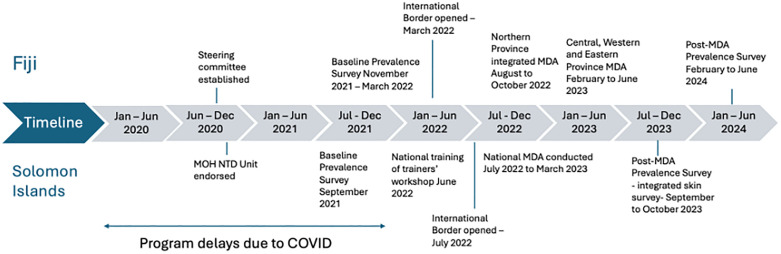
Implementation timeline for Fiji and Solomon Islands.

In Solomon Islands, the MDA commenced in July 2022 (Fig 3). The roll-out was led by provincial health authorities and each province took approximately four weeks to conduct the MDA campaign. MDA was implemented by nurses and volunteers from the province supported by boat drivers and local guides. Health promotion teams travelled with the MDA teams. The Solomon Islands program planned to integrate with a yaws MDA campaign that involved diagnostic screening and treatment with oral azithromycin, however, supplies for yaws MDA did not arrive in time for the MDA [21]. A NTD Unit was being formalized during this time which was instrumental in coordinating implementation. Prior to the NTD Unit, NTDs had been the mandate of a few health departments, for example, trachoma was under the Eye Care Department, deworming was under school health and scabies under dermatology. The formation of an NTD Unit has strengthened the ability to integrate the public health approaches needed for controlling NTDs.

In Fiji, the MDA commenced in the Northern Division in October 2022 (see timeline in Fig 3). The program integrated with the lymphatic filariasis program in the Northern and Eastern Divisions where lymphatic filariasis was co-endemic. In the Central and Western Divisions (the two larger divisions in Fiji), scabies MDA was not integrated with other NTDs; however, health teams used the opportunity to integrate with other health outreach programs, such as malnutrition screening. In Fiji, the MDA was implemented by teams of nurses, recent nurse graduates (employed as volunteers), and community health workers. Community awareness was conducted ahead of the MDA by engaging the traditional i-Taukei (indigenous Fijian) leadership, namely the Turaga-ni-Koro (village spokesman) and community health workers.

**Fig 4:**
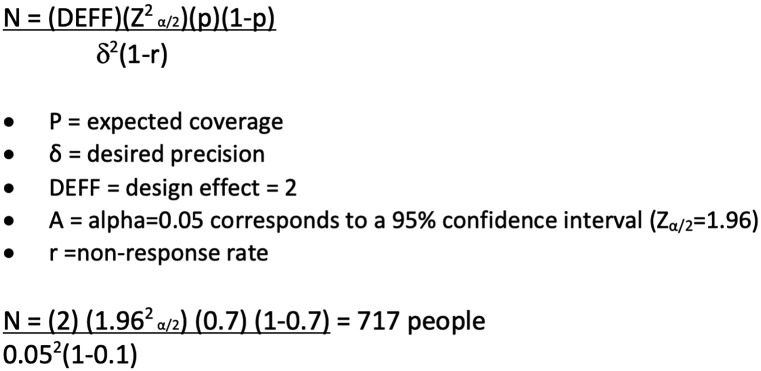
Coverage survey calculation for Fiji and Solomon Islands Coverage Surveys [22].

Coverage surveys were conducted after MDA in both countries, designed following WHO coverage guidance document [22]. The sample size was calculated as 717 community members using the formula outlined for coverage evaluation surveys in WHO guidance (Fig 4).

In Solomon Islands, six villages were randomly selected in 6 of the 10 provinces. In Fiji, ten villages were randomly selected in all four divisions. National program staff conducted coverage assessments by dividing each village into segments of approximately ten households. One segment was then randomly selected to be surveyed, with the intention of interviewing all members of the household, however, only those members present were interviewed.

In Solomon Islands, there were 1,077 respondents, and in Fiji, 882. The data was stratified by age category, province/division, and urban/peri-urban/rural. No weighting was applied. All data analysis was performed using Stata 18 (College Station, Texas, USA).

Ethics approval was not obtained for this work as this was a programmatic activity under Ministry of Health following normal consent processes and the coverage survey was under standard Ministry of Health monitoring activity. This article is not research but is based on the observations and lessons learned by World Scabies Program staff and Ministry of Health staff.

### Operational results

#### Solomon Islands.

Overall population coverage for the first dose of MDA was 49% (350,217 people), and 44% for second dose based on MDA registers. 50% of those receiving treatment were female. 86% received ivermectin, and 11% received permethrin cream due to ivermectin contraindication. 775 people refused MDA. The coverage survey estimated that 78% received the first dose of MDA, and 79% received the second dose. The survey estimated that 5% experienced mild side effects.

#### Fiji.

Overall population coverage for the first dose of MDA was 26% (231,351 people), and 18% for second dose. The coverage survey estimated that 45% received the first dose of MDA, and 23% received the second dose. The survey reported that 87% of respondents received ivermectin, and 13% permethrin cream, and 2% experienced mild side effects.

## Lessons learned

The Program encountered several challenges and identified several key learnings, that we categorized into the following themes: (1) expect the unexpected; (2) community perception of scabies; (3) integration with other NTD and health programs; (4) strategies for reaching urban populations; and (5) paediatric and single-dose formulations.

### Key learning 1: Expect the unexpected

Originally, the Program was scheduled to commence in 2020, however, the COVID-19 pandemic resulted in MDA implementation commencing two years later. The pandemic had several additional unexpected consequences for the program. First, as restrictions were lifted in each country other health programs hurried to implement, particularly routine immunization programs. As the same first-line staff are used for all health programs, this delayed commencement of the scabies MDA. Second, both countries had high attrition of health staff especially as both Australia and New Zealand actively recruited to fill vacancies in their aged care and health systems made apparent during the pandemic. In 2022, Fiji lost approximately 25% of their nurses (over 800 of 3,000 nurses) due to emigration to Australia or New Zealand [23]. Third, there was community reluctance to accepting visiting health teams as a result of misinformation disseminated with COVID-19 vaccines. Finally, the costs of logistics increased substantially during this time, particularly because of rising fuel costs associated with the conflict in Ukraine [24].

### Key learning 2: Community perceptions of scabies

**Fig 5:**
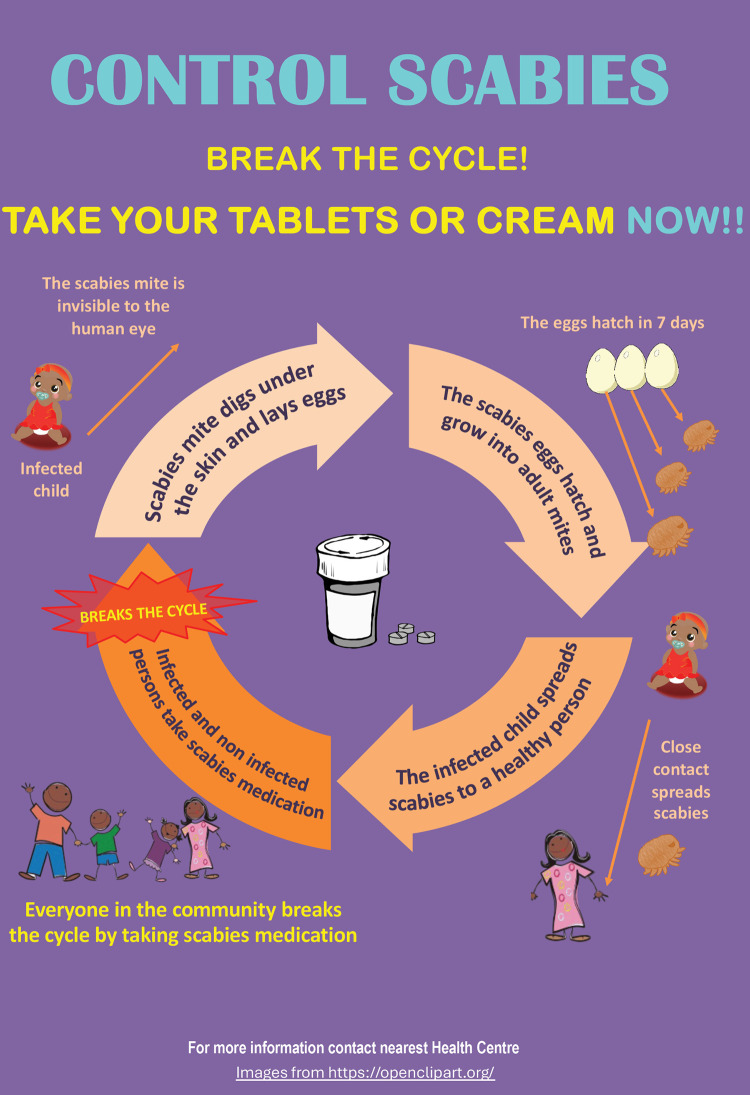
Poster in iTaukei explaining the MDA.

Scabies is common in both Fiji and Solomon Islands, and as such community members have experience of the disease over many generations. However, this has led to an array of misconceptions and local interpretations of the cause, transmission, risks, and treatment for scabies. A qualitative study in Fiji in 2019 observed limited understanding within many Fijian communities that scabies is caused by a mite, that it is spread from person-to-person and that it can lead to long-term consequences [25]. Some community members choose to use traditional medicines to treat scabies, and many see scabies as a childhood disease that is part of growing up and may not seek treatment at all. The study identified that community-based health promotion messaging on the social dynamics of scabies transmission is important to maximise success for control efforts in Fiji. In recognition of these findings, the Program designed community awareness messaging that sought to address misconceptions, such as explaining that scabies is caused by a mite, how scabies is transmitted through skin-to-skin contact, and directly addressing local misconceptions of scabies. The awareness program appealed to the strong community-minded culture in the Pacific Islands by emphasizing that taking part in MDA is not an individual choice but will help their family and broader community (see Fig 5).

Misunderstandings of scabies were not isolated to the lay community but were also noted among front-line health workers. We observed that in areas where healthcare workers understood the impact of scabies and the potential benefits of treating everyone in the community through MDA, healthcare staff were more motivated to ensure they reached all communities. During MDA training, it was apparent that some healthcare staff recommend treatments for scabies in their routine clinical practice inconsistent with standard treatment guidelines, such as the use of steroid creams. We identified a need to increase the knowledge and skills of nurses and community health workers through primary health care training on both the control and clinical management of scabies. Overall, we identified that improving general understanding of scabies among both the lay community and healthcare workers is important to the success of scabies MDA. These lessons will be factored into the next phase of the scabies control programs in Fiji and Solomon Islands. To ensure sustainability of the reduction in scabies following MDA, health systems strengthening measures that address WHO’s six building blocks for health systems will be implemented [26]. These measures will consider mainstreaming scabies care within health systems by training of the health workforce, increasing community awareness of scabies and its complications, strengthening supply chain management to ensure national availability of scabies medications and implementing surveillance protocols and systems to monitor the ongoing scabies case numbers.

### Key learning 3: Integration with other NTD and health programs

The Program sought to integrate the MDA scabies in both countries, however, was only successful in Fiji. There were multiple reasons why integrating the MDA with other health programs proved to be beneficial such as sharing costs of implementation and reducing the workload of healthcare workers. It also improved the acceptability of the program by senior management in health systems and by community members, especially as the COVID-19 pandemic delayed many critical routine health programs, such as immunization campaigns, as well as the scabies MDA. We found that Ministries of Health were more inclined to invest staff time in the scabies MDA if the program was also addressing other NTDs or other health priorities at the same time. Communities were also more receptive to integrated programs, preferring to address a number of health issues in one campaign, than have multiple health teams visiting.

While integration has the potential benefit of sharing of costs for logistics, community awareness, and staff, there are some challenges to overcome to achieve success. We found considerable time and investment were needed for joint planning and coordination. The Program identified several challenges related to timing of MDA around other planned program activities, product expiry dates, aligning procurement of supplies and materials, and dividing roles and responsibilities. In Fiji, the integration was across two different programs with different funding sources. As a result of competing program schedules, donor requirements, and potential product expiry dates, the two programs had to be creative in staffing and leadership of the campaigns to accommodate each programs’ other commitments. In Solomon Islands, planning for integration was facilitated by the presence of a NTD Unit. Nonetheless, integration of MDA implementation was challenging because of different donors with different priorities and late arrival of supplies, which meant integrated MDA for scabies and yaws did not go ahead [27].

The benefits of integration outweigh those of parallel programs, but require support from donors and senior management within the Ministry of Health to ensure all aspects are well coordinated and that implementation is not delayed.

### Key learning 4: Strategies for reaching urban populations

The Program anticipated that different strategies were needed to reach populations in urban areas due to diverse community structures, working habits, education levels, access to information, and experiences with healthcare services, however, the program was not always successful in addressing these challenges. We found that traditional strategies for community awareness such as using health promotion teams and house-to-house delivery of medicines were generally not effective for urban areas. This experience is consistent with that of MDA campaigns in the African context, where the core components of MDA (training, community awareness, delivery, and supervision) need to be adapted because of complex governance structures, population heterogeneity, mobility, and difficulties in building community trust in urban areas [28].

For community awareness, the use of radio and television in reaching urban populations appeared to be more effective than using health promotion teams. In Solomon Islands, community awareness was primarily conducted using health promotion teams which was successful in rural community settings such as Isabel and Makira (predominantly rural provinces) where coverage surveys found that 90% of respondents were aware of the MDA before dosing teams arrived in their community. This contrasted with Honiara (a province with large urban populations), where approximately half of respondents reported that they were informed about the MDA before the teams arrived. In Fiji, where radio and television advertisements were part of the community awareness strategy, one-third of the urban respondents reported hearing about the MDA through the media. Other methods of reaching urban communities, such as through religious leaders or other civil society groups, was not tried by the program but could be explored in future campaigns.

In both countries, lower coverage in urban areas was largely due to reach rather than refusal [29]. In Fiji, the coverage survey found that the two major reasons people did not take the medicines were because nobody came to their home (62%), or they were not home when the team came (30%). In the Honiara coverage survey, almost all respondents who did not take medicines reported that no one came to their home. We surmise that MDA teams may have visited these houses during the day when the occupants were at school or work.

To reach urban populations, we believe that NTD program delivery strategies need to be flexible and employ multiple approaches, such as setting up distribution sites at hubs of community activities including churches, markets, and schools to reach populations during the day. Our program was not able to utilize these methods due to a number of factors that included healthcare staff were reluctant to administer medicines in non-traditional settings such as churches, and parents were reluctant to allow dosing in schools (as they wanted to be present when their children were dosed due to concern for side effects). An additional approach to increase coverage in urban areas is for MDA teams to visit homes in the evening, however, again we found healthcare staff were reluctant to work after hours because of concerns for their safety when visiting homes after dark. More research and investment are required to understand how best to reach dense, urban areas with MDA, including exploration of micro-targeted MDA, an approach that has shown promise in some African contexts [28].

### Key learning 5: Paediatric and single-dose formulations

From a medicine treatment perspective, we identified two key barriers to the feasibility and effectiveness of scabies MDA: 1) the requirement to provide two doses of MDA medicine within two weeks; and 2) the lack of an oral paediatric formulation.

MDA for other NTDs typically consists of a single treatment, sometimes with multiple medicines, given either once or twice a year [30]. The cost, time, and logistics required to implement a two-dose MDA for scabies, reaching every community twice within two weeks, proved a barrier for the Program in Fiji and Solomon Islands. This approach was likely more costly than a one-dose strategy as it required additional staff and time to reach populations twice and required teams to carry larger volumes of medicine to provide two doses. It was logistically challenging to ensure the teams did not miss the 7–14-day window for the second dose. Both national programs had difficulty achieving coverage targets due to limitations of time and budget and were faced with challenges in planning and staffing. It was apparent to the Program that if the MDA was one dose instead of two, there would be several positive flow-on effects including increased available budget and staff for community awareness, greater supervision and oversight, more time to reach communities, and more room for innovation to reach different demographic groups including urban populations.

**Fig 6:**
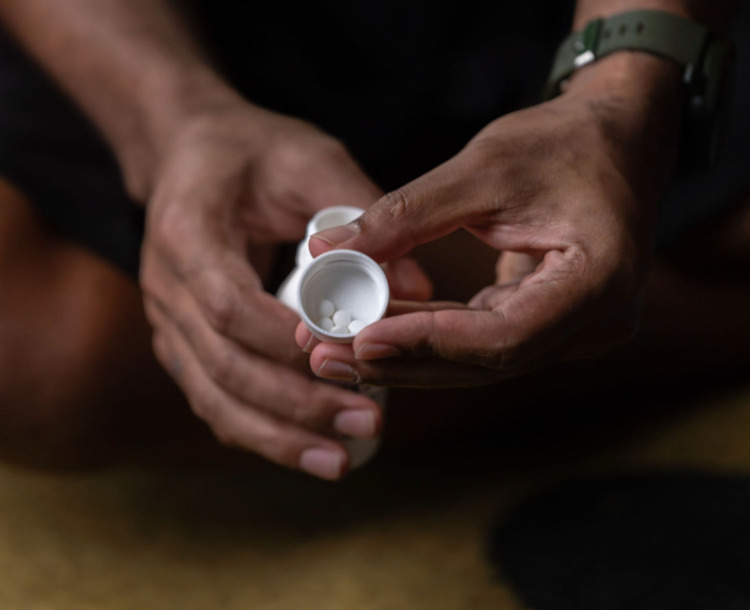
Ivermectin tablets.

**Fig 7:**
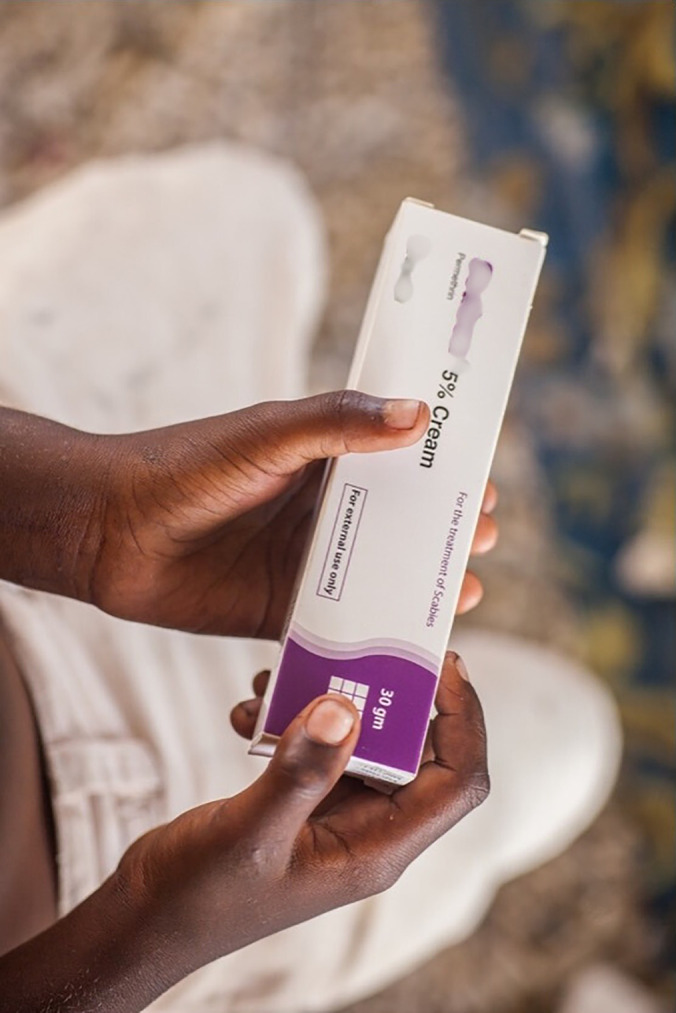
Permethrin cream.

Currently, ivermectin is not approved for use in children weighing less than 15 kg (or height less than 90 cm where height sticks are used) and permethrin cream is used in place of ivermectin for these small children. Permethrin cream is applied to the whole body and is washed off after 8 hours, with cream reapplied if someone washes their hands or accidentally washes the cream off some part of the body and children under the age of 2 months treated with one-quarter of a 30 g tube of permethrin and left on for 4 hours before being washed off. The child is not to bath or swim during that time. During MDA in Fiji and Solomon Islands, permethrin cream was provided with instructions for applying it at night, as this improves adherence to a longer duration of application. We found that a drawback of this approach was that parents were not directly observed applying the cream to their children, and so MDA teams could not ensure that directions were followed. Additionally, permethrin cream is far bulkier than ivermectin for transporting from the supplier to central stores and from village to village, and tube size and bulkiness increases transport costs and complicates logistics (Figs 6 and 7). Overall, we identified that providing effective treatment to young children through a paediatric appropriate oral medication would likely improve adherence and effectiveness of a scabies MDA. Promising research into the dosing and safety of ivermectin for children aged 2–4 years old has revealed that ivermectin is highly effective in treating scabies and well-tolerated [31]. Extending the use of ivermectin in this younger age group would likely increase the effectiveness of scabies control efforts. There are also new avermectins, such as moxidectin that have the potential to provide an effective oral, paediatric formulation [32].

## Conclusions

The implementation of scabies MDA in highly endemic areas has the potential to lead to substantial improvements in health outcomes. However, entire populations need to be reached to realise the benefits. Implementing scabies MDA on a large scale requires well-planned and well-funded community awareness, clear planning, and diverse strategies to reach urban populations. Integrating with other NTDs will be critical to the feasibility of MDA in contexts where scabies is highly prevalent and where there are many competing health needs. Finding an oral, paediatric treatment and a single-dose treatment option would improve the overall effectiveness of scabies MDA by improving coverage and adherence. The impact of the MDA campaigns in Fiji and Solomon Islands remains to be fully evaluated and will be published in future publications, but we have already learned a series of critical lessons to inform future efforts towards scabies control.

Many of these lessons outlined in this paper are particularly relevant for other contexts such countries in the Asia and Africa regions and the need for investments in medicine development and stronger urban strategies becomes more relevant in these more populous countries with large urban populations. Reaching populations in large African or Asian countries will require integration with other programs, and a single-dose, pediatric avermectin would enable scabies control treatments to be more easily integrated with deworming programs and immunization campaigns. Investing in finding cost-effective ways of reaching these populations with public health strategies for controlling scabies has the potential to provide major health benefits for endemic countries globally.
